# The top 100 most cited articles on mucopolysaccharidoses: a bibliometric analysis

**DOI:** 10.3389/fgene.2024.1377743

**Published:** 2024-04-12

**Authors:** Ruyu Liao, Rongrong Geng, Yue Yang, Yufan Xue, Lili Chen, Lan Chen

**Affiliations:** Department of Orthopedics, The Third People’s Hospital of Chengdu, Chengdu, China

**Keywords:** mucopolysaccharidoses, lysosomal storage disease, MPS, bibliometric analysis, VOSviewer

## Abstract

**Background:** Bibliometrics can trace general research trends in a particular field. Mucopolysaccharidoses (MPS), as a group of rare genetic diseases, seriously affect the quality of life of patients and their families. Scholars have devoted themselves to studying MPS’s pathogenesis and treatment modalities and have published many papers. Therefore, we conducted a bibliometric and visual study of the top 100 most highly cited articles to provide researchers with an indication of the current state of research and potential directions in the field.

**Methods:** The Web of Science Core Collection was searched for articles on MPS from 1 January 1900, to 8 November 2023, and the top 100 most cited articles were screened. The title, year of publication, institution, country, and first author of the articles were extracted and statistically analyzed using Microsoft Excel 2007. Keyword co-occurrence and collaborative networks were analyzed using VOSviewer 1.6.16.

**Results:** A total of 9,273 articles were retrieved, and the top 100 most cited articles were filtered out. The articles were cited 18,790 times, with an annual average of 188 citations (122–507). Forty-two journals published these articles, with Molecular Genetics and Metabolism and Proceedings of the National Academy of Sciences of the United States being the most published journal (N = 8), followed by Pediatrics (N = 7), Blood (N = 6). The United States (N = 68), the UK (N = 25), and Germany (N = 20) were the top contributing countries. The Royal Manchester Children’s Hospital (N = 20) and the University of North Carolina (N = 18) were the most contributing institutions. Muenzer J was the most prolific author (N = 14).

**Conclusion:** We conducted a bibliometric and visual analysis of the top 100 cited articles in MPS. This study identifies the most influential articles currently available in the field of MPS, which provides a good basis for a better understanding of the disease and informs future research directions.

## 1 Introduction

Mucopolysaccharidoses (MPSs) are a rare and heterogeneous group of inherited lysosomal storage disorders that can be classified into seven major disorders, including 11 subtypes (Kobayashi, 2019). The combined incidence of all MPS ranges from 1.53 to 4.8 cases per 100,000 live births and is characterized by progressive multiorgan involvement (Pinto et al., 2004; Khan et al., 2017). MPS is caused by defects in genes coding for different lysosomal enzymes degrading glycosaminoglycans (GAG), such as heparan sulfate (HS), chondroitin sulfate (CS), dermatan sulfate (DS) and keratan sulfate (KS). The deficient enzyme activity leads to systemic storage of GAG and a wide range of clinical manifestations (Puckett et al., 2021). For example, accumulation of GAG in growth plates and articular cartilage accelerates chondrocyte apoptosis and inflammation, leading to growth failure, limited joint range of motion, and reduced mobility (Clarke, 2011). Accumulation of GAG in the eye can lead to a variety of ocular comorbidities such as corneal clouding, glaucoma, retinopathy, and ocular nerve involvement, which can result in visual disability **(** Nagpal et al., 2022 **)**. There are also significant neurocognitive symptoms associated with MPS, such as developmental delays, behavioral disorders, and hydrocephalus (Shapiro and Eisengart, 2021). These disease manifestations seriously affect the quality of life of patients and their families. Scholars have devoted themselves to studying the pathogenesis and treatment modalities of MPS, exploring the efficacy and pitfalls of therapeutic modalities such as hematopoietic stem cell transplantation (HSCT), enzyme replacement therapy (ERT), and gene therapy (GT), and many papers have been published.

Citation analysis is essential to bibliometrics, identifying the most influential works in MPS(J. J. Zhou et al., 2017; Zhu et al., 2021). In general, the more citations an article has received, the more valuable and significant it is in the field (Kreutzer et al., 2017). Identifying the most cited works is crucial for clinicians or researchers in related fields to identify the most active areas and help guide future work. Therefore, this method is widely used in other areas of literature analysis (Karslı and Tekin, 2021; Liu et al., 2022) to identify high-quality articles in the field. However, few analyses have reported the most cited works on MPS. Hence, this study aimed to conduct a longitudinal review of the research in this field to provide a comprehensive picture of the research in the field and to identify the top 100 most cited articles on MPS in Web of science (WoS) in an effort to identify important contributions to the literature in the field as well as to provide direction for future research.

## 2 Materials and methods

### 2.1 Data sources

**FIGURE 1:**
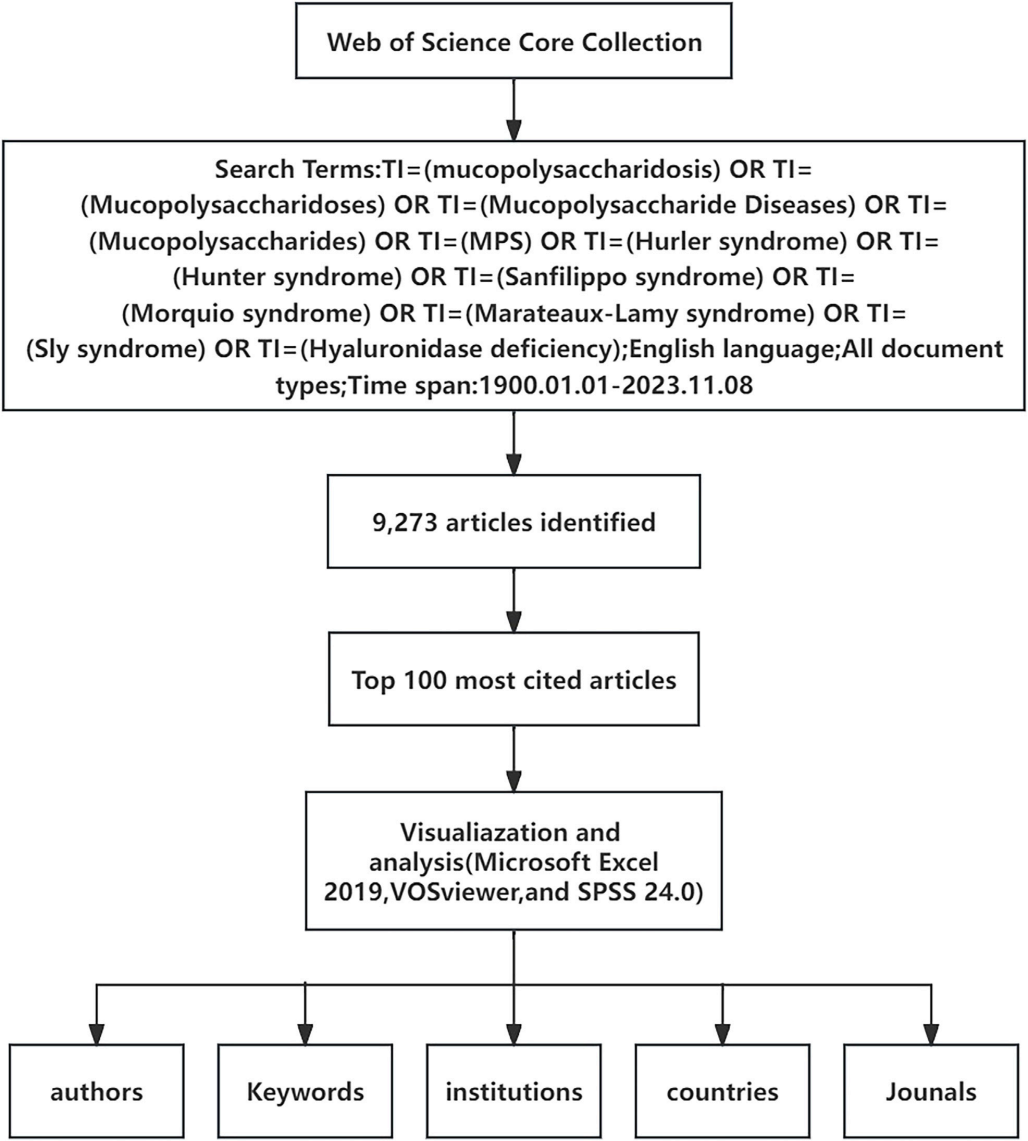
Flowchart of literature selection and analysis.

The number of citations of the same article in different databases is not the same, in order to avoid inconsistency in the results, so we choose only one database to search (Zhang et al., 2023). The Web of Science Core Collection (WoSCC) is the most extensively utilized database in academic research (L. Chen et al., 2023; Liu et al., 2024; Zhou et al., 2023), so we searched the WoSCC for articles related to MPS and sorted them in descending order of citations to filter the top 100 most-cited articles. The search was performed using the following terms: TI=(mucopolysaccharidosis) OR TI=(Mucopolysaccharidoses) OR TI=(Mucopolysaccharide Diseases) OR TI=(Mucopolysaccharides) OR TI=(MPS) OR TI=(Hurler syndrome) OR TI=(Hunter syndrome) OR TI=(Sanfilippo syndrome) OR TI=(Morquio syndrome) OR TI=(Marateaux-Lamy syndrome) OR TI=(Sly syndrome) OR TI=(Hyaluronidase deficiency), the language is set to English, the type of article is not limited, and the period from 1 January 1900 to 8 November 2023. Two investigators agreed on the search terms and independently screened the articles by reading the abstract or full text. If disagreements were encountered, a third researcher exercised judgment. This study did not require ethical approval as all data were obtained from publicly available WoS databases. (Figure 1).

### 2.2 Data extraction and organization

We extracted the following data from each article: title of the article, year of publication, first author, research institution and country (whichever is the first author), name of the journal in which the article was published, Journal Citation Reports (JCR) partition (if there are more than one partition, the highest division counts), impact factor, number of citations, type of article, average number of citations received after publication of each article, and WOS category (if it belongs to more than one category, the first one will be the most important). For the country information extracted from the study, we categorized Taiwan as China (Gao et al., 2019; Huang et al., 2023).

### 2.3 Statistical analysis

Descriptive statistical analysis of the articles was performed using Microsoft EXCEL 2007, containing title, year of publication, journal of publication, overall number of citations, average number of citations, impact factor, etc.; Correlation analysis was performed using SPSS 24.0, using the Pearson’s correlation coefficient (R) to determine that the difference was considered statistically significant when *p* < 0.05; Knowledge graphical analysis was performed using VOSviewer1.6.16 for knowledge graph analysis to map the collaborative network between countries, institutions and authors. The network contains three features: node size, connectivity, and color. A node represents a specific element such as country, author, or institution; the node’s size indicates the number or frequency of publications, and the node’s color indicates the year in which the article was published. The lines between nodes represent the number of times they appear together.

## 3 Result

### 3.1 Descriptive statistics

Based on the above search formula, we retrieved 9,273 articles related to MPS and filtered out the top 100 most cited documents.

### 3.2 Publication year, citation

**FIGURE 2:**
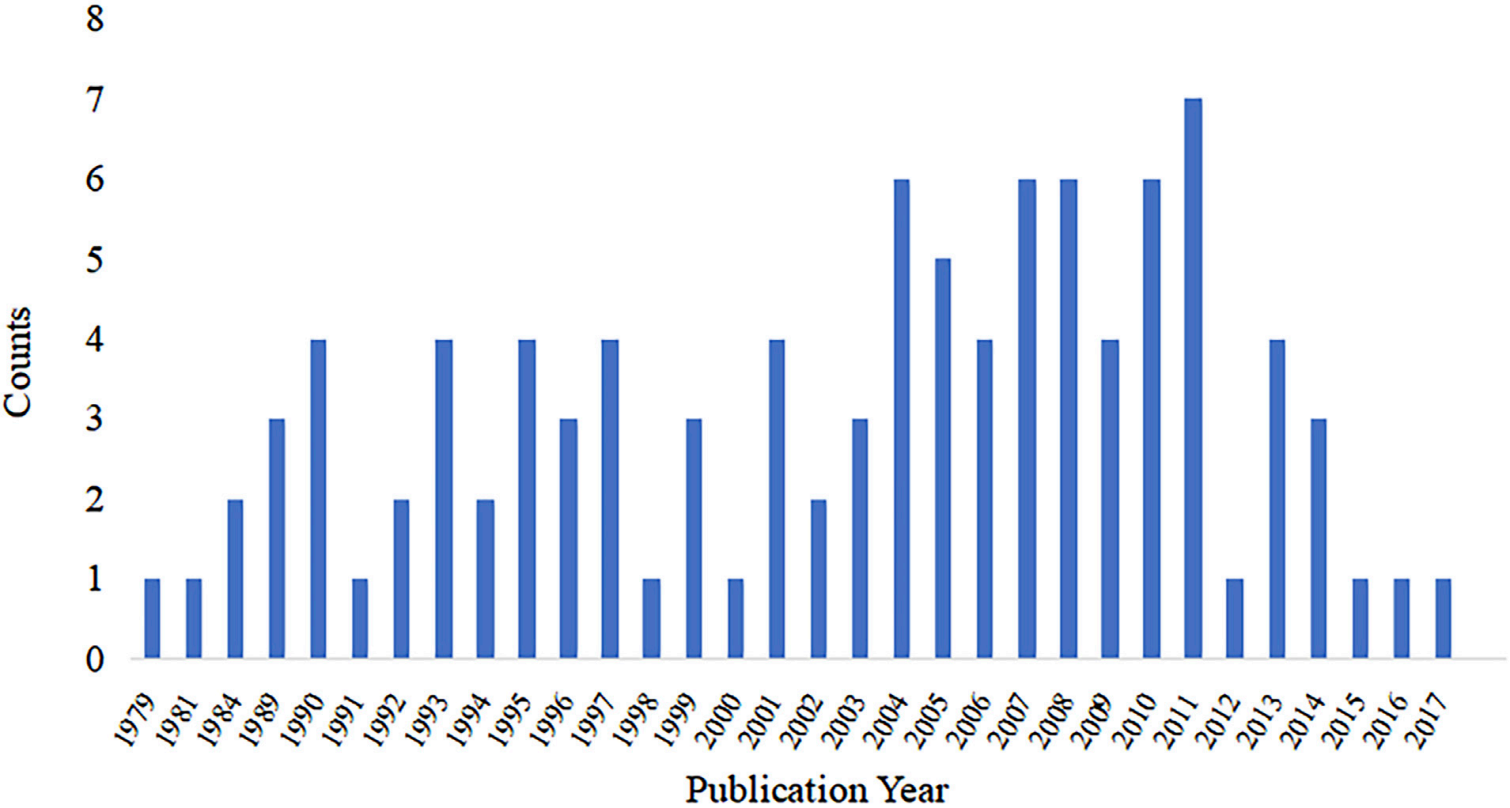
Publishing years of the 100 top-cited articles on mps.

Years of publication for the top 100 articles ranged from 1979 to 2017, articles published in 2018–2023 were not included. The annual publication rate varied from one to seven articles per year, with a majority of the articles (69%) being published since 1998. Notably, the highest number of articles (n = 7) was published in 2011. (Figure 2).

The top 100 articles were cited 18,790 times, with an annual average of 188 citations (122–507). There were 23 articles with more than 200 citations. A highly significant correlation existed between total citations and average annual citations (rs = 0.653, *p* < 0.001). There was no significant correlation between total citations and article age (rs = −0.007, *p* = 0.946). There was a significant correlation between average annual citations and article age (rs = −0.668, *p* < 0.001).

### 3.3 Article types and contents

The articles were ranked in descending order of citations to obtain the top 100 highly cited articles (Table 1). Among them, 85 were original articles and 15 were review articles.

**TABLE 1:** 100 top-cited articles on MPS.

Rank	First author	Article title	Journal	Publication year	Total citation	Average Annual Citation	Country
1	Kakkis, ED	Enzyme-Replacement Therapy in Mucopolysaccharidosis I	New England Journal of Medicine	2001	507	23	United States of America
2	Koç, ON	A Phase II/III Clinical Study of Enzyme	Bone Marrow Transplantation	2002	498	24	United States of America
		Allogeneic Mesenchymal Stem Cell Infusion for Treatment of Metachromatic Leukodystrophy (MLD) and Hurler Syndrome (MPS-IH)					
3	Muenzer, J	A Phase II/III Clinical Study of Enzyme Replacement Therapy with Idursulfase in Mucopolysaccharidosis II (Hunter Syndrome)	Genetics in Medicine	2006	442	26	United States of America
4	Wraith, JE	Enzyme Replacement Therapy for Mucopolysaccharidosis I: A Randomized, Double-Blinded, Placebo-Controlled, Multinational Study of Recombinant Human Α-L-Iduronidase (Laronidase)	Journal of Pediatrics	2004	427	22	United Kingdom
5	Snyder, EY	Neural Progenitor-Cell Engraftment Corrects Lysosomal Storage Throughout the MPS-VII Mouse-Brain	Nature	1995	398	14	United States of America
6	Staba, SL	Cord-blood transplants from unrelated donors in patients with Hurler’s syndrome	New England Journal of Medicine	2004	344	18	United States of America
7	Ohmi, K	Activated Microglia in Cortex of Mouse Models of Mucopolysaccharidoses I and IIIB	Proceedings of The National Academy of Sciences of The United States of America	2003	344	17	United States of America
8	Wraith, JE	Mucopolysaccharidosis Type II (Hunter Syndrome): A Clinical Review and Recommendations for Treatment in the Era of Enzyme Replacement Therapy	European Journal of Pediatrics	2008	331	22	United Kingdom
9	Muenzer, J	Mucopolysaccharidosis I: Management and Treatment Guidelines	Pediatrics	2009	315	23	United States of America
10	Muenzer, J	Overview of the Mucopolysaccharidoses	Rheumatology	2011	307	26	United States of America
11	Baehner, F	Cumulative Incidence Rates of the Mucopolysaccharidoses in Germany	Journal Of Inherited Metabolic Disease	2005	291	16	Germany
12	Harmatz, P	Enzyme Replacement Therapy for Mucopolysaccharidosis VI: A Phase 3, Randomized, Double-Blind, Placebo-Controlled, Multinational Study of Recombinant Human N-Acetylgalactosamine 4-Sulfatase (Recombinant Human Arylsulfatase B or Rhasb) And Follow-On, Open-Label Extension Study	Journal Of Pediatrics	2006	287	17	United States of America
13	Birkenmeier, EH	Murine Mucopolysaccharidosis Type-VII - Characterization of A Mouse with Beta-Glucuronidase Deficiency	Journal of Clinical Investigation	1989	272	8	United States of America
14	Valstar, MJ	Sanfilippo Syndrome: A Mini-Review	Journal of Inherited Metabolic Disease	2008	256	17	Netherlands
15	Peters, C	Hurler Syndrome: II. Outcome of HLA Genotypically Identical Sibling And HLA-Haploidentical Related Donor Bone Marrow Transplantation in Fifty-Four Children	Blood	1998	256	10	United States of America
16	Wilson, PJ	Hunter Syndrome - Isolation of An Iduronate-2-Sulfatase Cdna Clone	Proceedings of the National Academy of Sciences of the United States of America	1990	249	8	Australia
		And Analysis of Patient DNA					
17	Peters, C	Outcome of Unrelated Donor Bone	Blood	1996	247	9	United States of America
		Marrow Transplantation in 40 Children with Hurler Syndrome					
18	Clarke, LA	Long-Term Efficacy and Safety of Laronidase in the Treatment of Mucopolysaccharidosis I	Pediatrics	2009	245	18	Canada
19	Valayannopoulos, V	Mucopolysaccharidosis VI	Orphanet Journal of Rare Diseases	2010	223	17	France
20	Aldenhoven, M	Long-Term Outcome of Hurler Syndrome Patients after Hematopoietic Cell Transplantation: An International Multicenter Study	Blood	2015	217	27	Netherlands
21	Martin, R	Recognition and Diagnosis of Mucopolysaccharidosis II (Hunter Syndrome)	Pediatrics	2008	214	14	United States of America
22	Wolfe, JH	Reversal of Pathology in Murine	Nature	1992	211	7	United States of America
		Mucopolysaccharidosis Type-VII By Somatic-Cell Gene-Transfer					
23	Harmatz, P	Enzyme Replacement Therapy in Mucopolysaccharidosis VI (Maroteaux-Lamy Syndrome)	Journal of Pediatrics	2004	210	11	United States of America
24	Wraith, JE	Enzyme Replacement Therapy in Patients Who Have Mucopolysaccharidosis I and Are Younger Than 5 Years: Results of A Multinational Study of Recombinant Human Α-L-Iduronidase (Laronidase)	Pediatrics	2007	194	12	United Kingdom
25	Ashworth, JL	Mucopolysaccharidoses and the Eye	Survey of Ophthalmology	2006	191	11	United Kingdom
26	Tardieu, M	Intracerebral Administration of Adeno-Associated Viral Vector Serotype Rh.10 Carrying Human SGSH and SUMF1 cDNAs in Children with Mucopolysaccharidosis Type IIIA Disease: Results of A Phase I/II Trial	Human Gene Therapy	2014	189	21	France
27	Whitley, CB	Diagnostic-Test for Mucopolysaccharidosis .1. Direct Method for Quantifying Excessive Urinary Glycosaminoglycan Excretion	Clinical Chemistry	1989	182	5	United States of America
28	Giugliani, R	Management Guidelines for Mucopolysaccharidosis VI	Pediatrics	2007	181	11	United States of America
29	Moullier, P	Correction of Lysosomal Storage in The Liver and Spleen of MPS-VII Mice by Implantation of Genetically-Modified Skin Fibroblasts	Nature Genetics	1993	181	6	FRANCE
30	Sifuentes, M	A Follow-Up Study of MPS I Patients Treated with Laronidase Enzyme Replacement Therapy for 6 Years	Molecular Genetics and Metabolism	2007	180	11	United States of America
31	Sands, MS	Enzyme Replacement Therapy for Murine Mucopolysaccharidosis Type-VII	Journal of Clinical Investigation	1994	180	6	United States of America
32	Sokolov, EP	An Improved Method for DNA Isolation from Mucopolysaccharide-Rich Molluscan Tissues	Journal of Molluscan Studies	2000	179	8	Russia
33	Braunlin, EA	Cardiac Disease in Patients with Mucopolysaccharidosis: Presentation, Diagnosis and Management	Journal of Inherited Metabolic Disease	2011	178	15	United States of America
34	Mcglynn, R	Differential Subcellular Localization of Cholesterol, Gangliosides, and Glycosaminoglycans in Murine Models of Mucopolysaccharide Storage Disorders	Journal of Comparative Neurology	2004	177	9	United States of America
35	Muenzer, J	The Mucopolysaccharidoses: A Heterogeneous Group of Disorders with Variable Pediatric Presentations	Journal of Pediatrics	2004	175	9	United States of America
36	Birkenmeier, EH	Increased Life-Span and Correction of Metabolic Defects in Murine Mucopolysaccharidosis Type-VII After Syngeneic Bone-Marrow Transplantation	Blood	1991	174	5	United States of America
37	Nelson, J	Incidence of the Mucopolysaccharidoses in Northern Ireland	Human Genetics	1997	173	7	Australia
38	Simonaro, CM	Mechanism of Glycosaminoglycan-Mediated Bone and Joint Disease - Implications for the Mucopolysaccharidoses and Other Connective Tissue Diseases	American Journal of Pathology	2008	169	11	United States of America
39	Li, HH	Mouse Model of Sanfilippo Syndrome Type B Produced by Targeted Disruption of the Gene Encoding Α-N-Acetylglucosaminidase	Proceedings of the National Academy of Sciences of the United States of America	1999	169	7	United States of America
40	Dejong, JGN	Dimethylmethylene Blue-Based Spectrophotometry of Glycosaminoglycans in Untreated Urine - A Rapid Screening-Procedure for Mucopolysaccharidoses	Clinical Chemistry	1989	169	5	NETHERLANDS
41	Harmatz, P	Long-Term Follow-Up of Endurance and Safety Outcomes During Enzyme Replacement Therapy for Mucopolysaccharidosis Vi: Final Results of Three Clinical Studies of Recombinant Human N-Acetylgalactosamine 4-Sulfatase	Molecular Genetics and Metabolism	2008	168	11	United States of America
42	Hopwood, JJ	The Mucopolysaccharidoses - Diagnosis, Molecular-Genetics and Treatment	Molecular Biology and Medicine	1990	167	5	Australia
43	Muenzer, J	A Phase I/II Clinical Trial of Enzyme Replacement Therapy in Mucopolysaccharidosis II (Hunter Syndrome)	Molecular Genetics and Metabolism	2007	165	10	United States of America
44	Harmatz, P	Direct Comparison of Measures of Endurance, Mobility, And Joint Function During Enzyme-Replacement Therapy of Mucopolysaccharidosis VI (Maroteaux-Lamy Syndrome): Results After 48 Weeks in A Phase 2 Open-Label Clinical Study of Recombinant Human N-Acetylgalactosamine 4-Sulfatase	Pediatrics	2005	163	9	United States of America
45	Sango, K	Mice Lacking both Subunits of Lysosomal Beta-Hexosaminidase Display Gangliosidosis and Mucopolysaccharidosis	Nature Genetics	1996	163	6	United States of America
46	Wolfe, JH	Herpesvirus Vector Gene-Transfer and Expression of Beta-Glucuronidase in The Central-Nervous-System of MPS-VII Mice	Nature Genetics	1992	163	5	United States of America
47	De Ru, MH	Enzyme Replacement Therapy and/or Hematopoietic Stem Cell Transplantation at Diagnosis in Patients with Mucopolysaccharidosis Type I: Results of A European Consensus Procedure	Orphanet Journal of Rare Diseases	2011	162	14	Netherlands
48	Fu, HY	Correction of Neurological Disease of Mucopolysaccharidosis IIIB in Adult Mice by Raav9 Trans-Blood-Brain Barrier Gene Delivery	Molecular Therapy	2011	161	13	United States of America
49	Muenzer, J	Long-Term, Open-Labeled Extension Study of Idursulfase in The Treatment of Hunter Syndrome	Genetics in Medicine	2011	161	13	United States of America
50	Haskins, ME	Beta-Glucuronidase Deficiency in A Dog - A Model of Human Mucopolysaccharidosis-VII	Pediatric Research	1984	159	4	United States of America
51	Haurigot, V	Whole Body Correction of Mucopolysaccharidosis IIIA by Intracerebrospinal Fluid Gene Therapy	Journal of Clinical Investigation	2013	158	16	Spain
52	Nelson, J	Incidence of the Mucopolysaccharidoses in Western Australia	American Journal of Medical Genetics Part A	2003	157	8	Australia
53	Boelens, JJ	Outcomes of Hematopoietic Stem Cell Transplantation for Hurler’s Syndrome in Europe: A Risk Factor Analysis for Graft Failure	Bone Marrow Transplantation	2007	155	10	Netherlands
54	Tomatsu, S	Mucopolysaccharidosis Type Iva (Morquio A Disease): Clinical Review and Current Treatment: A Special Review	Current Pharmaceutical Biotechnology	2011	154	13	United States of America
55	Voznyi, YV	A Fluorimetric Enzyme Assay for The Diagnosis of MPS II (Hunter Disease)	Journal of Inherited Metabolic Disease	2001	154	7	Netherlands
56	Scott, HS	Molecular Genetics of Mucopolysaccharidosis Type I: Diagnostic, Clinical, and Biological Implications	Human Mutation	1995	154	6	Australia
57	Krivit, W	Bone-Marrow Transplantation in the Maroteaux-Lamy Syndrome (Mucopolysaccharidosis Type-VI) - Biochemical and Clinical Status 24 Months after Transplantation	New England Journal of Medicine	1984	154	4	United States of America
58	Triggs-Raine, B	Mutations In Hyal1, A Member of A Tandemly Distributed Multigene Family Encoding Disparate Hyaluronidase Activities, Cause A Newly Described Lysosomal Disorder, Mucopolysaccharidosis Ix	Proceedings of the National Academy of Sciences of the United States of America	1999	152	6	Canada
59	Whitley, CB	Long-Term Outcome of Hurler Syndrome Following Bone-Marrow Transplantation	American Journal of Medical Genetics	1993	152	5	United States of America
60	Piotrowska, E	Genistein-Mediated Inhibition of Glycosaminoglycan Synthesis as A Basis for Gene Expression-Targeted Isoflavone Therapy for Mucopolysaccharidoses	European Journal of Human Genetics	2006	151	9	Poland
61	Vandekamp, JJP	Genetic-Heterogeneity and Clinical Variability in The Sanfilippo Syndrome (Type-A, Type-B, And Type-C)	Clinical Genetics	1981	151	4	NETHERLANDS
62	Vogler, C	Overcoming the Blood-Brain Barrier with High-Dose Enzyme Replacement Therapy in Murine Mucopolysaccharidosis VII	Proceedings of the National Academy of Sciences of the United States of America	2005	149	8	United States of America
63	Bhaumik, M	A Mouse Model for Mucopolysaccharidosis Type Iii A (Sanfilippo Syndrome)	Glycobiology	1999	149	6	United States of America
64	Kakkis, ED	Long-Term and High-Dose Trials of Enzyme Replacement Therapy in the Canine Model of Mucopolysaccharidosis	Biochemical and Molecular Medicine	1996	149	6	United States of America
65	Khan, SA	Epidemiology of Mucopolysaccharidoses	Molecular Genetics and Metabolism	2017	147	25	United States of America
66	Souillet, G	Outcome of 27 Patients with Hurler’s Syndrome Transplanted from Either Related or Unrelated Haematopoietic Stem Cell Sources	Bone Marrow Transplantation	2003	147	7	France
67	Visigalli, I	Gene Therapy Augments the Efficacy of Hematopoietic Cell Transplantation and Fully Corrects Mucopolysaccharidosis Type I Phenotype in the Mouse Model	Blood	2010	146	11	Italy
68	Pastores, GM	The MPS I Registry: Design, Methodology, and Early Findings of A Global Disease Registry for Monitoring Patients with Mucopolysaccharidosis Type I	Molecular Genetics and Metabolism	2007	146	9	United States of America
69	Vellodi, A	Bone Marrow Transplantation for Mucopolysaccharidosis Type I: Experience of Two British Centres	Archives of Disease in Childhood	1997	146	6	United Kingdom
70	Sands, MS	Treatment of Murine Mucopolysaccharidosis Type-VII by Syngeneic Bone-Marrow Transplantation in Neonates	Laboratory Investigation	1993	146	5	United States of America
71	Vogler, C	A Murine Model of Mucopolysaccharidosis-VII - Gross and Microscopic Findings in Beta-Glucuronidase-Deficient Mice	American Journal of Pathology	1990	146	4	United States of America
72	Boelens, JJ	Outcomes of Transplantation Using Various Hematopoietic Cell Sources in Children with Hurler Syndrome after Myeloablative Conditioning	Blood	2013	145	15	Netherlands
73	Clarke, LA	Murine Mucopolysaccharidosis Type I: Targeted Disruption of the Murine Alpha-L-Iduronidase Gene	Human Molecular Genetics	1997	145	6	Canada
74	Hendriksz, CJ	Efficacy And Safety of Enzyme Replacement Therapy with Bmn 110 (Elosulfase Alfa) for Morquio A Syndrome (Mucopolysaccharidosis Iva): A Phase 3 Randomised Placebo-Controlled Study	Journal of Inherited Metabolic Disease	2014	143	16	United Kingdom
75	Simonaro, CM	Involvement of the Toll-Like Receptor 4 Pathway and Use of TNF-Α Antagonists for Treatment of the Mucopolysaccharidoses	Proceedings of the National Academy of Sciences of the United States of America	2010	140	11	United States of America
76	Mcgill, JJ	Enzyme Replacement Therapy for Mucopolysaccharidosis VI from 8 Weeks of Age-A Sibling Control Study	Clinical Genetics	2010	139	11	Australia
77	Lin, HY	Incidence of the Mucopolysaccharidoses in Taiwan, 1984–2004	American Journal of Medical Genetics Part A	2009	139	10	China
78	Kakkis, E	Intrathecal Enzyme Replacement Therapy Reduces Lysosomal Storage in the Brain and Meninges of The Canine Model of MPS I	Molecular Genetics and Metabolism	2004	139	7	United States of America
79	Ponder, KP	Therapeutic Neonatal Hepatic Gene Therapy in Mucopolysaccharidosis VII Dogs	Proceedings of the National Academy of Sciences of the United States of America	2002	139	7	United States of America
80	Wilkinson, FL	Neuropathology In Mouse Models of Mucopolysaccharidosis Type I, IIIA and IIIB	Plos One	2012	138	13	United Kingdom
81	Shull, RM	Enzyme Replacement in A Canine Model of Hurler-Syndrome	Proceedings of the National Academy of Sciences of the United States of America	1994	138	5	United States of America
82	Valstar, MJ	Mucopolysaccharidosis Type IIIA: Clinical Spectrum and Genotype-Phenotype Correlations	Annals of Neurology	2010	137	11	Netherlands
83	Taylor, RM	Decreased Lysosomal Storage in the Adult MPS VII Mouse Brain in the Vicinity of Grafts of Retroviral Vector-Corrected Fibroblasts Secreting High Levels of Beta-Glucuronidase	Nature Medicine	1997	136	5	United States of America
84	Wraith, JE	The Mucopolysaccharidoses - A Clinical Review and Guide to Management	Archives of Disease in Childhood	1995	136	5	United Kingdom
85	Cleary, MA	Management of Mucopolysaccharidosis Type-III	Archives of Disease in Childhood	1993	135	5	United Kingdom
86	Bondeson, ML	Inversion of the Ids Gene Resulting from Recombination with Ids-Related Sequences is A Common-Cause of The Hunter Syndrome	Human Molecular Genetics	1995	132	5	SWEDEN
87	Muenzer, J	Multidisciplinary Management of Hunter Syndrome	Pediatrics	2009	131	9	United States of America
88	Keeling, KM	Gentamicin-Mediated Suppression of Hurler Syndrome Stop Mutations Restores A Low Level of Α-L-Iduronidase Activity and Reduces Lysosomal Glycosaminoglycan Accumulation	Human Molecular Genetics	2001	131	6	United States of America
89	Vandiggelen, OP	A Fluorimetric Enzyme Assay for the Diagnosis of Morquio-Disease Type-A (MPS-Iv-A)	Clinica Chimica Acta	1990	131	4	NETHERLANDS
90	Muenzer, J	Early Initiation of Enzyme Replacement Therapy for the Mucopolysaccharidoses	Molecular Genetics and Metabolism	2014	130	14	United States of America
91	Hopwood, JJ	A Fluorometric Assay Using 4-Methylumbelliferyl Alpha-L-Iduronide for The Estimation of Alpha-L-Iduronidase Activity and the Detection of Hurler and Scheie Syndromes	Clinica Chimica Acta	1979	130	3	United Kingdom
92	Scarpa, M	Mucopolysaccharidosis Type II: European Recommendations for the Diagnosis and Multidisciplinary Management of A Rare Disease	Orphanet Journal of Rare Diseases	2011	129	11	United Kingdom
93	Hendriksz, CJ	Review of Clinical Presentation and Diagnosis of Mucopolysaccharidosis Iva	Molecular Genetics and Metabolism	2013	126	13	United Kingdom
94	Aldenboven, M	The Clinical Outcome of Hurler Syndrome after Stem Cell Transplantation	Biology of Blood and Marrow Transplantation	2008	126	8	Netherlands
95	Tomatsu, S	Mutation and Polymorphism Spectrum of The Galns Gene in Mucopolysaccharidosis Iva (Morquio A)	Human Mutation	2005	126	7	United States of America
96	Simonaro, CM	Joint and Bone Disease in Mucopolysaccharidoses Vi and VII: Identification of New Therapeutic Targets and Biomarkers Using Animal Models	Pediatric Research	2005	126	7	United States of America
97	Yogalingam, G	Molecular Genetics of Mucopolysaccharidosis Type IIIA and IIIB: Diagnostic, Clinical, and Biological Implications	Human Mutation	2001	126	6	Australia
98	Scott, CR	Identification of Infants at Risk for Developing Fabry, Pompe, or Mucopolysaccharidosis-I from Newborn Blood Spots by Tandem Mass Spectrometry	Journal of Pediatrics	2013	124	12	United States of America
99	Giugliani, R	Mucopolysaccharidosis I, II, And VI: Brief Review and Guidelines for Treatment	Genetics and Molecular Biology	2010	124	10	Brazil
100	Muenzer, J	A Phase I/II Study of Intrathecal Idursulfase-It in Children with Severe Mucopolysaccharidosis II	Genetics in Medicine	2016	122	17	United States of America

**FIGURE 3:**
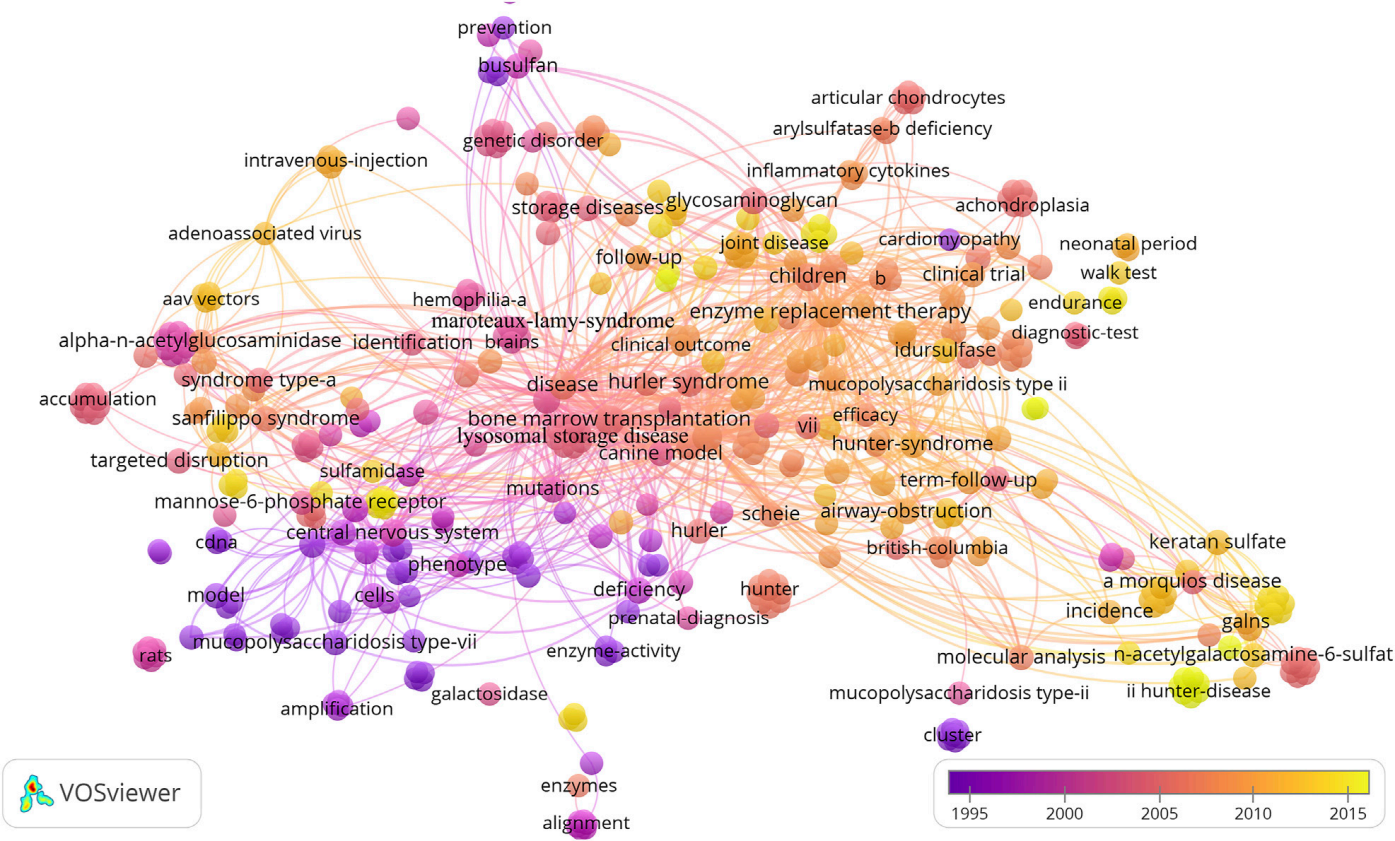
The co-occurrence network of keywords network on MPS.

We found these articles mainly focused on the epidemiology, drug treatment trials, animal experiments, identification and diagnosis, management, and treatment guidelines of MPS by reading the titles or abstracts. These articles belong to 17 categories of Web of Science, of which the top three are Genetics and Heredity (N = 18), Pediatrics (N = 18), and Endocrinology and Metabolism (N = 13) (Table 2). Bone marrow transplantation (BMT), enzyme replacement therapy, lysosomal storage disease, hurler syndrome, hunter-syndrome, and central nervous system (CNS) were the high-frequency keywords that appeared (Figure 3).

**TABLE 2:** Type of study and categories in the 100 top-cited studies on MPS.

Variable	Number of studies	Total citation times	Average citation time per study
Type of study			
Article	85	16102	189
Review	15	2688	179
Web of Science categories^a^			
Genetics and Heredity	18	3063	170
Pediatrics	18	3699	206
Endocrinology and Metabolism	13	2223	171
Multidisciplinary Sciences	11	2227	202
Biochemistry and Molecular Biology	10	1438	144
Hematology	7	1311	187
Medical Laboratory Technology	4	612	153
Medicine, Research and Experimental	4	756	189
Biophysics	3	800	267
Medicine, General and Internal	3	1005	335
Biotechnology and Applied Microbiology	2	350	175
Pathology	2	315	158
Clinical Neurology	1	137	137
Marine and Freshwater Biology	1	179	179
Neurosciences	1	177	177
Ophthalmology	1	191	191
Rheumatology	1	307	307

^a^ Web of Science categories were identified from web of science, if one article was listed in more than one category, the first category was used for data analysis.

### 3.4 Journal analysis

Forty-two journals published these articles; Table 3 shows the top 10 journals with more than three publications. Of these, Molecular Genetics and Metabolism and Proceedings of the National Academy of Sciences of the United States of America was the most published journal (N = 8), followed by Pediatrics (N = 7). The IF of 42 journals varied from 1.2 to 158.5. There were 22 journals with an IF < 5.000, 11 from 5.000–10.000, nine with an IF > 10.000, and three journals with an IF > 40. Three journals were not included in the 2022 edition of the JCR. The journal with the highest IF (158.5) was the New England Journal of Medicine, which published three of the most cited articles. Nine of the top 15 journals in the JCR are in Q1, five are in Q2, and one is in Q3.

**TABLE 3:** Journals publishing the top 100 most cited articles.

Journal	Quartile in category	Number of publications	Total citations	Citations per article	If (2022)	Jif quartile
Proceedings of the National Academy of Sciences of The United States of America	Multidisciplinary Sciences	8	1480	185	11.1	Q1
Molecular Genetics and Metabolism	Endocrinology and Metabolism	8	1201	150	3.8	Q2
Pediatrics	Pediatrics	7	1443	206	8.0	Q1
Blood	Hematology	6	1185	198	20.3	Q1
Journal of Inherited Metabolic Disease	Endocrinology and Metabolism	5	1022	204	4.2	Q2
Journal of Pediatrics	Pediatrics	5	1223	245	3.6	Q1
New England Journal of Medicine	Medicine, General and Internal	3	1005	335	158.5	Q1
Nature Genetics	Genetics and Heredity	3	507	169	30.8	Q1
Journal of Clinical Investigation	Medicine, Research and Experimental	3	610	203	15.9	Q1
Genetics In Medicine	Genetics and Heredity	3	725	242	8.8	Q1
Archives of Disease in Childhood	Pediatrics	3	417	139	5.2	Q1
Bone Marrow Transplantation	Hematology	3	800	267	4.8	Q2
Human Mutation	Genetics and Heredity	3	406	135	3.9	Q2
Orphanet Journal of Rare Diseases	Genetics and Heredity	3	514	171	3.7	Q2
Human Molecular Genetics	Biochemistry and Molecular Biology	3	403	134	3.5	Q3

### 3.5 Analysis of country

**FIGURE 4:**
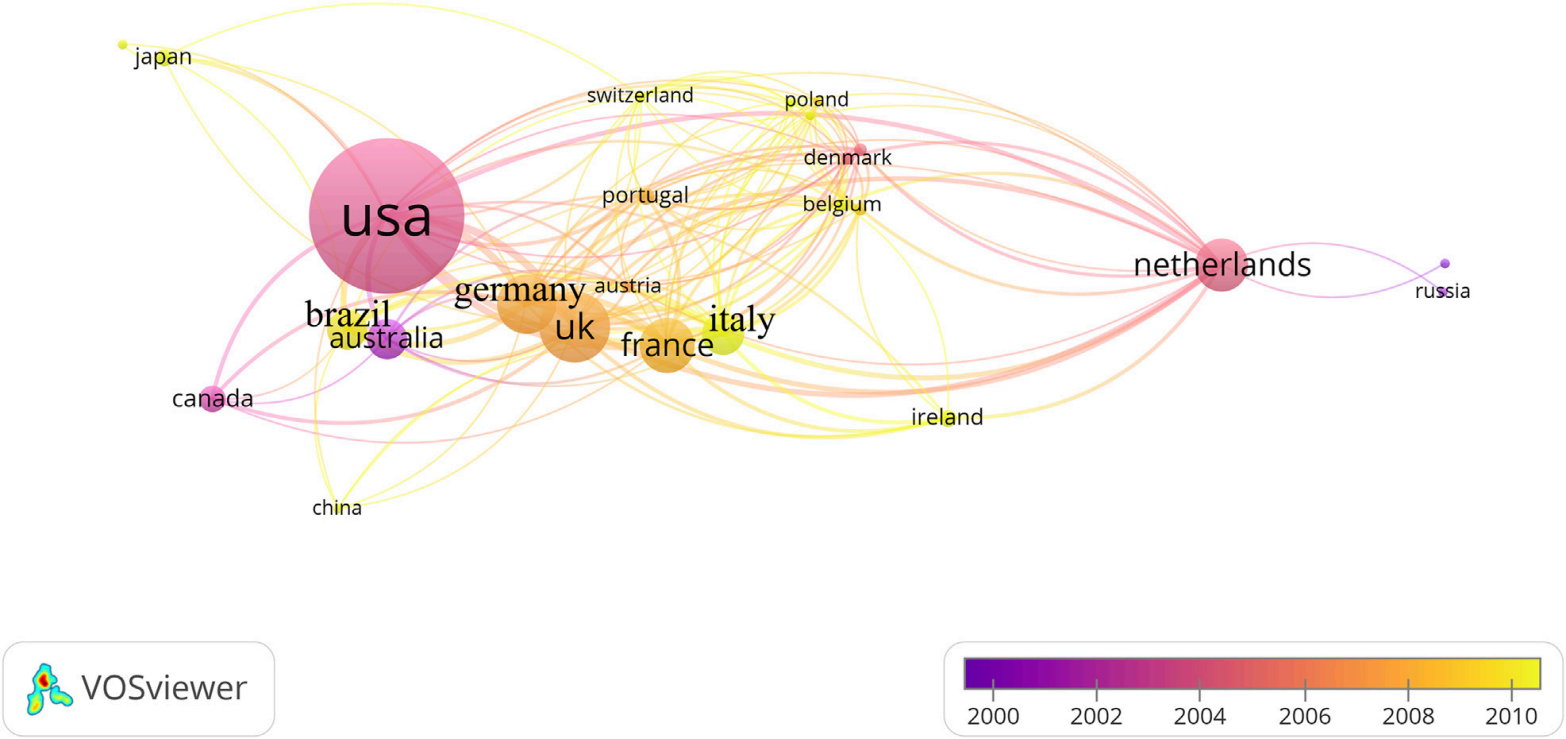
The country collaboration network on MPS.

A total of 25 countries published these 100 papers. Table 4 shows the top ten countries with the most publications. Among the top 100 most cited articles, the USA (N = 68) contributed the most, followed by the UK(N = 25) and Germany (N = 20). When ranked by the average number of citations per article, the top three are Canada (226), Germany (212), and the UK (199). A vast network of collaborations has been formed in this field, with the United States of America, UK, and Germany having very close collaborations (Figure 4).

**TABLE 4:** Top 10 countries contributing to the 100 most cited articles.

Rank	Country	Number of articles	Total citations	Mean citations per article
1	United States of America	68	13380	197
2	United Kingdom	25	4967	199
3	Germany	20	4247	212
4	France	18	3280	182
5	Netherlands	17	2896	170
6	Italy	13	2381	183
7	Australia	12	2163	180
9	Brazil	12	2043	170
8	Canada	7	1584	226
10	Ireland	4	679	170

### 3.6 Analysis of institution

**FIGURE 5:**
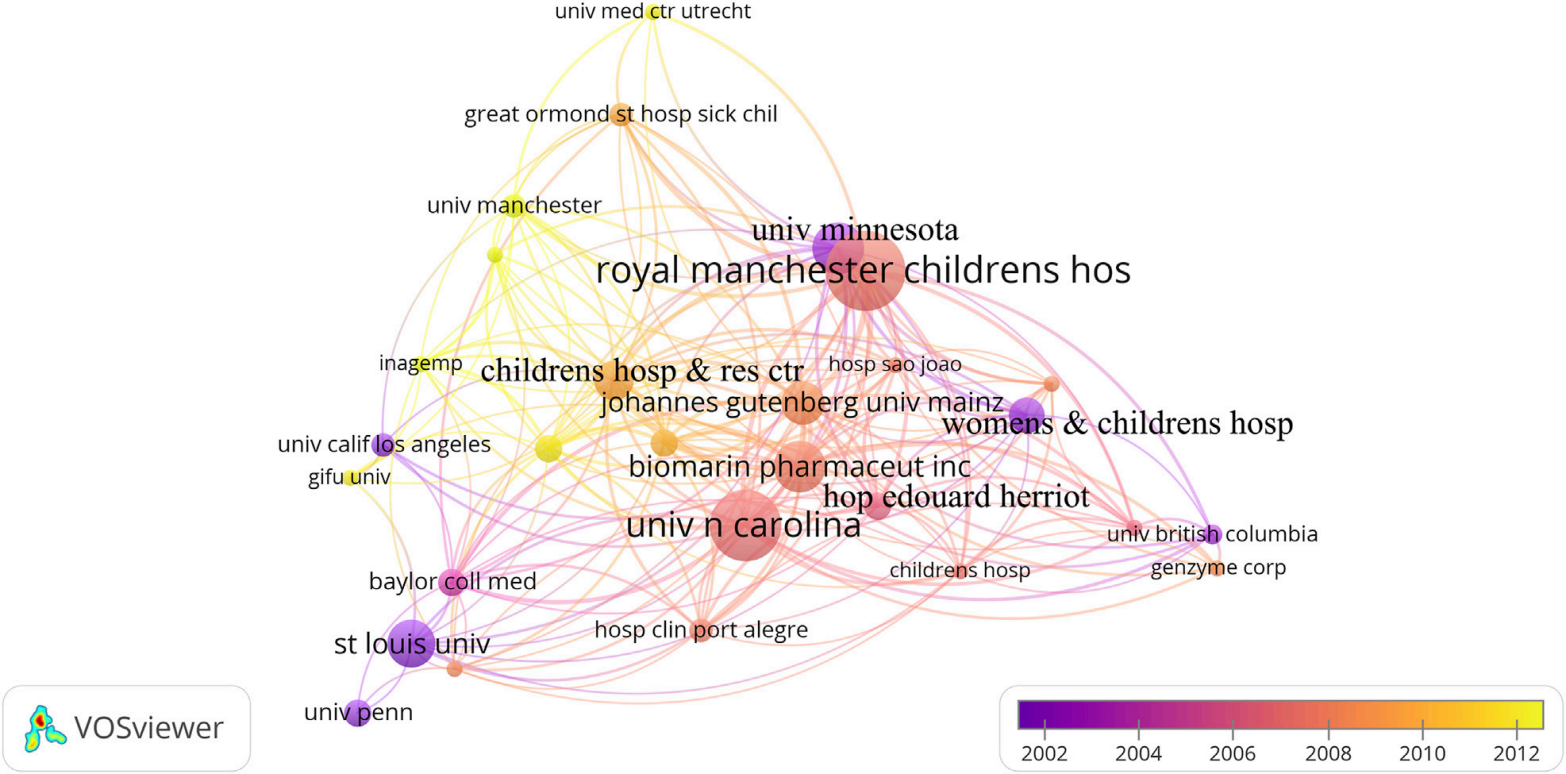
The institution collaboration network on MPS.

A total of 234 institutions contributed to the one hundred articles. Table 5 shows the top 10 institutions contributing seven or more articles, six from the United States of America. The most significant contributor was Royal Manchester Children’s Hospital (N = 20) from the UK, followed by the University of North Carolina with 18 articles, Biomarin Pharmaceut Inc. and the University of Minnesota both with 13 articles. Led by the top institutions, the institutions collaborated extensively and closely, forming a more extensive collaborative network (Figure 5).

**TABLE 5:** Institutions contributing to the 100 most cited articles.

Rank	Institution	Country	Number of article	Total citations	Mean citations per article
1	Royal Manchester Children’s Hospital	United Kingdom	20	4315	216
2	University of North Carolina	United States of America	18	4341	241
3	Biomarin Pharmaceut Inc.	United States of America	13	2941	226
4	the University of Minnesota	United States of America	13	2844	219
5	Saint Louis University	United States of America	12	2042	170
6	Johannes Gutenberg Universita¨t mainz	Germany	11	2560	233
7	Children’s Hospital and Research Center	United States of America	10	2072	207
8	Womens and Childrens Hospital	Australia	9	1602	178
9	Baylor College of Medicine	United States of America	7	1803	258
10	Hôpital Edouard Herriot	France	7	1236	177

### 3.7 Analysis of author

**FIGURE 6:**
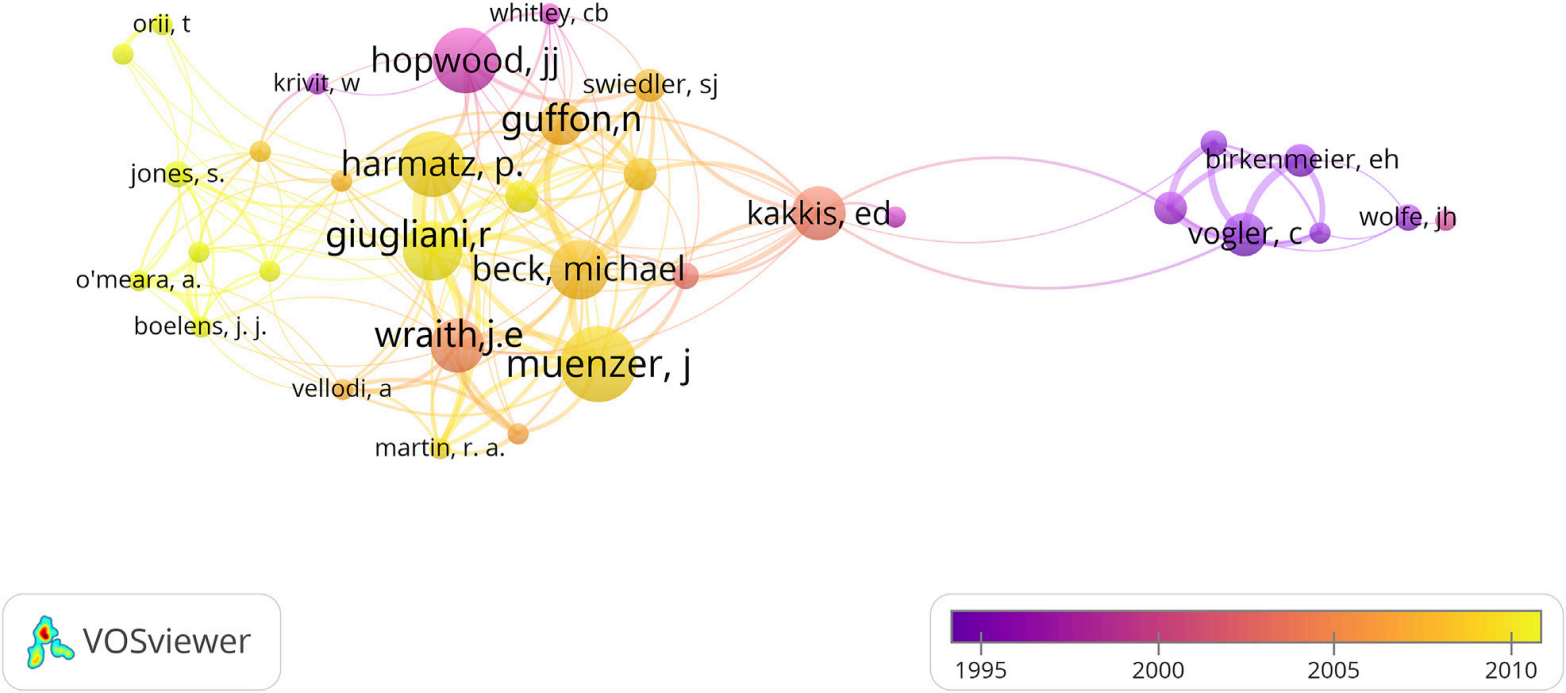
The author collaboration network on MPS.

557 authors contributed to 100 articles, and Table 6 shows the top 10 authors who contributed the most to these 100 articles. Muenzer J was the most prolific author, with 14 publications and 3,487 citations. This scholar mainly focused on treating MPS type II, i.e., Hunter’s syndrome (Muenzer et al., 2006; Muenzer et al., 2007; Muenzer, 2014). This was followed by Harmatz, P (N = 12) and Hopwood, JJ (N = 12). Muenzer, J and Harmatz, P are both from the United States of America, and Hopwood, JJ is from Australia. After visually analyzing author collaborations using VOSviewer and plotting the knowledge graph several times, the minimum number of author appearances was set to four (Figure 6). Most researchers do not appear in our graph because they have fewer than four articles. The nodes in the graph represent authors, and the larger the node, the greater the number of articles they have published. Extensive collaboration exists between most of the top authors.

**TABLE 6:** The top 10 authors most frequently appearing in publications.

Rank	Author	Affiliation	Country	Number of articles	Total citations	Mean citations per article
1	Muenzer, J	University of North Carolina	United States of America	14	3487	249
2	Harmatz, P	University of California San Francisco	United States of America	12	2449	204
3	Hopwood, JJ	South Australian Health and Medical Research Institute	Australia	12	2130	178
4	Beck, M	University Medical Center of Mainz; Johannes Gutenberg Universita¨t mainz	Germany	11	2906	264
5	Giugliani, R	Universidade Federal do Rio Grande do Sul; Hospital de Clínicas de Porto Alegre	Brazil	11	2215	201
6	Kakkis, ED	Ultragenyx Pharmaceutical Inc.	United States of America	10	2415	242
7	Wraith, J. ED	Royal Manchester Children’s Hospital	United Kingdom	10	2236	224
8	Guffon, N	Center References Malad Hereditaires Metab; Hôpital Edouard Herriot	France	8	1567	196
9	Vogler, C	Saint Louis University	United States of America	8	1417	177
10	Birkenmeier, EH	Jackson Laboratory	United States of America	6	1129	188

## 5 Discussion

This study reviews clinical and research advances by bibliometric and visual mapping of the top 100 most cited articles in the field of MPS, with the expectation of providing new ideas to researchers. Molecular Genetics and Metabolism and Proceedings of the National Academy of Sciences of the United States of America published the highest number of papers, and the New England Journal of Medicine published the most articles with the highest average number of citations. The United States was the most productive country. The Royal Manchester Children’s Hospital was the most influential institution. Muenzer J was the most prolific author, with 14 publications. This study found that there is extensive and close collaboration between the top-ranked countries, institutions, and authors, and analyzing these collaborative networks not only visualizes the number of publications, but also reflects their connections and the evolution and development of the field as a whole, and it can help us to retrieve resources more efficiently (Liu et al., 2024).

Among the top 100 most cited articles, 27 are MPS I (11 of them on MPS IH), 11 are MPS II, nine are MPS III (four on MPS IIIA), five are MPS IV (all on MPS IVA), eight are MPS VI, three are MPS VII, one is MPS IX, and the rest of the articles do not have a clear classification of the types of MPS were not categorized. As we can see, among the 100 most cited articles, MPS I is the most popular type. The possible reason for this is that MPS I is the most common type of MPS, with a higher prevalence than the other types, and therefore there is more attention paid to it (Scott et al., 1995; Çelik et al., 2021). MPS II is the first MPS disease to be reported, and the manifestations of this disease were described in detail by Dr. Hunter in 1917 (Hunter, 1917), hence the name Hunter syndrome. However, the treatment of MPS lagged by decades. In 1968, a study by Elizabeth Neufeld et al. first found that MPS progression could be delayed or even terminated by providing deficient enzymes to MPS patients (Fratantoni et al., 1968). This result provided the framework for the modern treatment of MPS. Research in this field has been going on for more than a hundred years, but the amount of research produced is far less than the short burst of Covid19-related research (Y. Chen et al., 2021; Wang et al., 2023; Zhang et al., 2022). There are several possible reasons for this: MPS is a rare disease with a low incidence, and thus may attract less research attention (Platt, 2018); The lack of public awareness of MPS may affect the raising of research funds and the promotion of research; Due to the limited number of MPS patients, there are few clinical data available for research, which may limit the depth of research and the development of new treatment methods. Therefore, increasing the understanding of MPS and investing more research resources are of great significance to improve the diagnosis and treatment of MPS patients.

Keywords are the condensed summary of an article, and if they frequently appear together, they are considered to be a research hotspot in this field (Liu et al., 2024; Zhu and Zhang, 2021). The co-occurrence analysis of keywords in this study showed that bone marrow transplantation, enzyme replacement therapy, lysosomal storage disease, hurler syndrome, Children, alpha-l-iduronidase, hunter-syndrome, and central nervous system were the research hotspots in the field of MPS. These keywords mainly included the classification and treatment of MPS. Lysosomal storage disease (LSD) is a group of inherited metabolic diseases that includes more than 70 diseases (Parenti et al., 2021), of which MPS is a subclass. The earliest attempts to treat LSD were to use bone marrow transplantation (BMT) in patients with MPS I, also known as hematopoietic stem cell transplantation (HSCT). The success of such attempts has resulted in hundreds of patients benefiting from this treatment and extending their life expectancy (Aldenhoven et al., 2015; Rodgers et al., 2017; Taylor et al., 2019; Guffon et al., 2021). In addition, HSCT has a good effect on improving neurocognitive function. Therefore, it is also still considered a first-line treatment for MPS IH, even though it requires frequent medical interventions and creates a substantial burden of disease (Taylor et al., 2019). The findings of long-term studies and the implementation of management guidelines on enzyme replacement therapy (ERT) suggest that patients with MPS derive multiple benefits from this treatment (Giugliani et al., 2007; Muenzer et al., 2009; Muenzer et al., 2011a; Muenzer et al., 2011b; Hendriksz et al., 2014; Hendriksz et al., 2016). The most cited review article (Wraith et al., 2008) and Randomised controlled trial (RCT) article (Muenzer et al., 2006) both reported the therapeutic effect of idursulfase replacement therapy for MPS II, a weekly infusion of idursulfase (0.5 mg/kg) could significantly increase walking distance, improve lung function, increase elbow range of motion, reduce urinary GAG levels, and reduce organ size in patients with MPS II. However, conventional idursulfase does not cross the blood-brain barrier and may not improve CNS dysfunction in patients with severe MPS II. Therefore, a new generation of ERT has been developed and studied to overcome the inability of conventional ERT to reach the CNS, which will be described later.

Among the top 100 articles, there are 45 basic studies and 31 clinical studies. Basic research is the cornerstone of research in the biomedical field, and the etiology, pathogenesis, and treatment methods of MPS(Baldiotti et al., 2021). The most cited basic studies (Snyder et al., 1995) published in Nature in 1995, which transplanted p-glucuronidase-expressing neural progenitor cells into the ventricles of MPS VII neonatal mice and showed that lysosomal stores were significantly reduced or absent in both neurons and glial cells of treated MPS VII mice compared to untreated controls. This provides a model for using neural progenitor cells to transfer other foreign genes or factors to the CNS. Recent basic studies have shed light on the link between storage-related substances, lysosomal dysfunction, innate immune activation, and hyperinflammation that aggravate MPS symptoms, and these mechanisms could be important targets for new therapies (Kendall and Holian, 2021; Tillo et al., 2022; Xu and Núñez, 2023). Therefore, a new generation of ERT has been developed and investigated to overcome the problem that conventional ERT therapy does not reach the CNS, which we will talk about later.

The United States of America was the most prolific country, publishing 68 percent of the highly cited articles, followed by the UK, Germany, and France, with the majority of the top 100 articles coming from Europe and the United States, with only one coming from Asia. The contribution of the United States is reported to be influential not only in the field of MPS but also in other fields such as orthopedics. On the one hand, this is because the United States of America has many top academic institutions and scientific research personnel (Adnan and Ullah, 2018). On the other hand, the United States provides strong support and more funding for academic activities (Bullock et al., 2018; da Costa Rosa et al., 2022), which provides a solid foundation for academic research. Canada, the UK, and Germany are all important research partners, and these countries are also highly productive in the field, forming a close-knit collaborative network among themselves. However, when we changed the metric to the average number of citations per article, the top three countries became Canada, Germany, and the UK. We believe that one reason for this is that all three countries have close collaborations with the United States, and their research findings have increased visibility and dissemination, so their research findings are likely to receive more citations (Sugimoto et al., 2017; Chinchilla-Rodríguez et al., 2019). The second reason may be that they publish fewer articles and have a more focused area of research, which makes them more likely to be cited. The larger size of the research community in the United States may result in a wider distribution of citations for many articles, thus reducing the average citation rate. although the average citation index per article is higher, this does not necessarily reflect a country’s overall research output or impact. Therefore we need to consider a variety of factors when selecting indicators for evaluation.

In general, rare diseases rarely attract the attention of pharmaceutical companies due to their small number of patients, complex conditions, and high research and development costs (Platt, 2018). Interestingly, however, some pharmaceutical companies were included in our study and were among the top 100 highest-yielding institutions. Such as Genzyme corp., Biomarin pharmaceutics inc. Both companies specialize in the development of drugs for rare diseases. Genzyme Corp. developed the first biological therapy for LSD, enzyme replacement therapy for Gaucher disease type 1 (Brady, 2006). This is an achievement of academic and commercial co-creation that has yielded promising clinical results and improved clinical outcomes for patients with Gaucher disease. Since then, Genzyme has focused on rare diseases, developing enzyme replacement drugs for patients with LSD to improve their quality of life (Clarke et al., 2009; Muenzer et al., 2011a). Genzyme corp’s product Aldurazyme™ can significantly improve the respiratory function and joint movement of patients with MPS Ⅰ, reduce the accumulation of glycosaminoglycan, and has good safety. Naglazyme™ developed by Biomarin pharmaceutics inc can significantly improve joint movement, valvular heart disease, and scoliosis in patients (McGill et al., 2010). Therefore, the development of medicine cannot be done without the active involvement of pharmaceutical companies.

The impact factor represents the frequency with which a journal has been cited over some time and is an important measure of a journal’s academic impact (Mainwaring et al., 2020). The highest impact factor in this study was the New England Journal of Medicine, with an IF of 158.5. The second and third-ranked journals were Nature Medicine and Nature, with ifs of 82.9 and 64.8, respectively. These top journals attract a large number of high-quality papers, which in turn are published by these journals to further increase their academic impact (Callaham et al., 2002). Interestingly, Molecular Genetics and Metabolism (N = 8), IF = 3.8, one of the journals that published the most cited papers in this study, had an IF = 3.8, which suggests that even low IF journals can have highly cited papers and that we should pay attention to the quality of the papers and the value of the research itself as a real contribution to the field (Duan et al., 2022). In addition, the lower IF of journals focusing exclusively on metabolic diseases may be due to the smaller population studying these rare diseases. Thus, the lower IF does not reflect the importance of journals such as Molecular Genetics and Metabolism for metabolic diseases.

The number of citations of an article is related to multiple factors, such as IF, publication time, and accessibility of the journal (Zhu et al., 2021). Typically, the IF represents the quality and impact of a journal’s articles (Karsan et al., 2019). The most cited article in this study was published in the New England Journal of Medicine. In addition to the importance of the research results, the IF of the journal may also be the reason for its high citation. Our analysis found no significant correlation between the total number of citations and the age of articles, that is, articles published later may receive more citations, which is similar to the results of (Zhu et al., 2021). An article by Khan, SA et al. published in 2017 (Khan et al., 2017) was published in a short period but ranked third in average annual citations (N = 25). This indicates that this article has played an important guiding role in the research in this field, and it can be predicted that it will become a new article with a high impact in the future. In addition, paid journals may have fewer citations than open-access journals, because some readers are not willing to pay for access to article resources, so they choose to look for the same type of article in open-access journals instead, resulting in fewer citations.

In recent years, with the joint efforts of scholars all over the world, some promising treatments have emerged in the field of MPS.

Pabinafusp alfa (JR-141), a novel ERT drug developed in Japan, can cross the blood-brain barrier through transferrin receptor transcytoendocytosis and has shown positive results in clinical trials and been successfully approved for marketing (Giugliani et al., 2021; Okuyama et al., 2021). The study showed a significant reduction in GAG accumulation in the cerebrospinal fluid of patients with MPS II, indicating successful delivery of pabinafusp alfa with favorable clinical outcomes. This is potentially valuable for patients with MPS accompanied by CNS disease.

*In vivo* gene therapy is a promising option. The safety and tolerability of intracerebral administration of AAVrh.10 vectors carrying the human SGSH gene with the PGK promoter have been demonstrated in four patients with MPSIIIA (Tardieu et al., 2014). Another piece of good news is that Regenxbio announces a pivotal trial of RGX-121 for the treatment of MPS II achieves the primary endpoint, patients with reduced cerebrospinal fluid biomarkers below maximum attenuated disease levels (*p* = 0.00016) (Regenxbio, 2024). RGX-121 has also been previously reported to consistently reduce GAGs in CSF(Regenxbio, 2023), with some patients still benefiting for up to 3 years (Regenxbio, 2023). *Ex vivo* HSCGT also has great potential in the treatment of MPS disorders, has proven revolutionary in similar lysosomal disorders, and is currently in several clinical trials (Wood and Bigger, 2022).

Several immunomodulatory drugs have also been used in the treatment of MPS and are promising. In 2017, Polgreen et al. conducted a clinical study of adalimumab (a human monoclonal antibody that blocks TNF-α), which showed that adalimumab may help to reduce pain and improve physical and neurological function in patients with MPS I and II (Polgreen et al., 2017). Anakinra is a recombinant, non-glycosylated human interleukin-1 receptor antagonist, which can improve neurocognitive symptoms when used in MPS III patients (NCT 04018755). Resveratrol is a natural phenolic compound and phytoantitoxin, and Rintz et al. demonstrated that long-term continuous administration of 50 mg/kg/day of resveratrol improved neurological symptoms and reduced urinary GAG levels in a mouse model of MPS IIIB (Rintz et al., 2023). The application of these treatments is very promising in the future, and scholars can do more exploration based on the above results.

Increased knowledge of MPS’s pathophysiology and natural history and therapeutic modalities such as HSCT and ERT have improved survival and reduced morbidity. However, there are still some issues that need to be addressed, such as the safety of gene therapy, expensive treatments, and bone deformity (Donati et al., 2018). In addition to new treatments, the disease diagnosis should be moved forward. For example, newborn screening associated with MPS is increasingly being implemented. But before that, more comprehensive epidemiologic investigations of patients with MPS are needed to provide a basis for determining appropriate newborn screening methods. If managed appropriately, this should lead to earlier initiation of treatment and better outcomes. We also hope that more attention and resources will be devoted to research on MPS and other rare diseases to bring patients longer and better lives.

## 6 Limitation

This study has several limitations. First, the data in this study came from the WoS core repository, and articles from other databases, such as PubMed and Scopus were not searched, which may lead to some missing research results. Second, the citation counts in this study did not exclude self-citations, which may also lead to bias in the results, with some high-impact articles having fewer citations instead. Some articles may have been cited more often because they have been open for a more extended period, which does not represent the quality of the articles. Third, the quality of the top 100 articles was not assessed in this study, so it is possible that there are articles of varying quality, affecting the interpretation of the results. Last and most importantly, although we reviewed articles in this field, we did not include influential or highly cited papers published in the last 5 years, and new developments in this field are not reflected in our article. We will analyze the latest developments in this field in a subsequent article.

## 7 Conclusion

We conducted a bibliometric and visual analysis of the top 100 cited articles in MPS, a rich and promising area of research. This study identifies the most influential articles currently available in the field of MPS, which provides a good basis for a better understanding of the disease and informs future research directions.

## Data Availability

The original contributions presented in the study are included in the article/Supplementary material, further inquiries can be directed to the corresponding author.

## References

[B1] Adnan S. Ullah R. (2018). Top-cited articles in regenerative endodontics: a bibliometric analysis. J. Endod. 44 (11), 1650–1664. 10.1016/j.joen.2018.07.015 30243658

[B2] Aldenhoven M. Wynn R. F. Orchard P. J. O'Meara A. Veys P. Fischer A. (2015). Long-term outcome of Hurler syndrome patients after hematopoietic cell transplantation: an international multicenter study. Blood 125 (13), 2164–2172. 10.1182/blood-2014-11-608075 25624320

[B3] Baldiotti A. L. P. Amaral-Freitas G. Barcelos J. F. Freire-Maia J. Perazzo M. F. Freire-Maia F. B. (2021). The top 100 most-cited papers in cariology: a bibliometric analysis. Caries Res. 55 (1), 32–40. 10.1159/000509862 33341798

[B4] Brady R. O. (2006). Enzyme replacement for lysosomal diseases. Annu. Rev. Med. 57, 283–296. 10.1146/annurev.med.57.110104.115650 16409150

[B5] Bullock N. Ellul T. Bennett A. Steggall M. Brown G. (2018). The 100 most influential manuscripts in andrology: a bibliometric analysis. Basic Clin. Androl. 28, 15. 10.1186/s12610-018-0080-4 30564366 PMC6290538

[B6] Callaham M. Wears R. L. Weber E. (2002). Journal prestige, publication bias, and other characteristics associated with citation of published studies in peer-reviewed journals. JAMA 287 (21), 2847–2850. 10.1001/jama.287.21.2847 12038930

[B7] Çelik B. Tomatsu S. C. Tomatsu S. Khan S. A. (2021). Epidemiology of mucopolysaccharidoses update. Diagn. (Basel) 11 (2), 273. 10.3390/diagnostics11020273 PMC7916572 33578874

[B8] Chen L. Wan Y. Yang T. Zhang Q. Zeng Y. Zheng S. (2023). Bibliometric and visual analysis of single-cell sequencing from 2010 to 2022. Front. Genet. 14, 1285599. 10.3389/fgene.2023.1285599 38274109 PMC10808606

[B9] Chen Y. Zhang X. Chen S. Zhang Y. Wang Y. Lu Q. (2021). Bibliometric analysis of mental health during the COVID-19 pandemic. Asian J. Psychiatr. 65, 102846. 10.1016/j.ajp.2021.102846 34562753 PMC8435062

[B10] Chinchilla-Rodríguez Z. Sugimoto C. R. Larivière V. (2019). Follow the leader: on the relationship between leadership and scholarly impact in international collaborations. PLoS One 14 (6), e0218309. 10.1371/journal.pone.0218309 31220123 PMC6586445

[B11] Clarke L. A. (2011). Pathogenesis of skeletal and connective tissue involvement in the mucopolysaccharidoses: glycosaminoglycan storage is merely the instigator. Rheumatol. Oxf. 50 (5), v13–v18. 10.1093/rheumatology/ker395 22210665

[B12] Clarke L. A. Wraith J. E. Beck M. Kolodny E. H. Pastores G. M. Muenzer J. (2009). Long-term efficacy and safety of laronidase in the treatment of mucopolysaccharidosis I. Pediatrics 123 (1), 229–240. 10.1542/peds.2007-3847 19117887

[B13] da Costa Rosa T. Pintor A. V. B. Magno M. B. Marañón-Vásquez G. A. Maia L. C. Neves A. A. (2022). Worldwide trends on molar incisor and deciduous molar hypomineralisation research: a bibliometric analysis over a 19-year period. Eur. Arch. Paediatr. Dent. 23 (1), 133–146. 10.1007/s40368-021-00676-5 34674159

[B14] Donati M. A. Pasquini E. Spada M. Polo G. Burlina A. (2018). Newborn screening in mucopolysaccharidoses. Ital. J. Pediatr. 44 (2), 126. 10.1186/s13052-018-0552-3 30442156 PMC6238254

[B15] Duan S. L. Qi L. Li M. H. Liu L. F. Wang Y. Guan X. (2022). The top 100 most-cited papers in pheochromocytomas and paragangliomas: a bibliometric study. Front. Oncol. 12, 993921. 10.3389/fonc.2022.993921 36185194 PMC9523535

[B16] Fratantoni J. C. Hall C. W. Neufeld E. F. (1968). Hurler and Hunter syndromes: mutual correction of the defect in cultured fibroblasts. Science 162 (3853), 570–572. 10.1126/science.162.3853.570 4236721

[B17] Gao Y. Shi S. Ma W. Chen J. Cai Y. Ge L. (2019). Bibliometric analysis of global research on PD-1 and PD-L1 in the field of cancer. Int. Immunopharmacol. 72, 374–384. 10.1016/j.intimp.2019.03.045 31030093

[B18] Giugliani R. Harmatz P. Wraith J. E. (2007). Management guidelines for mucopolysaccharidosis VI. Pediatrics 120 (2), 405–418. 10.1542/peds.2006-2184 17671068

[B19] Giugliani R. Martins A. M. So S. Yamamoto T. Yamaoka M. Ikeda T. (2021). Iduronate-2-sulfatase fused with anti-hTfR antibody, pabinafusp alfa, for MPS-II: a phase 2 trial in Brazil. Mol. Ther. 29 (7), 2378–2386. 10.1016/j.ymthe.2021.03.019 33781915 PMC8261166

[B20] Guffon N. Pettazzoni M. Pangaud N. Garin C. Lina-Granade G. Plault C. (2021). Long term disease burden post-transplantation: three decades of observations in 25 Hurler patients successfully treated with hematopoietic stem cell transplantation (HSCT). Orphanet J. Rare Dis. 16 (1), 60. 10.1186/s13023-020-01644-w 33517895 PMC7847591

[B21] Hendriksz C. J. Burton B. Fleming T. R. Harmatz P. Hughes D. Jones S. A. (2014). Efficacy and safety of enzyme replacement therapy with BMN 110 (elosulfase alfa) for Morquio A syndrome (mucopolysaccharidosis IVA): a phase 3 randomised placebo-controlled study. J. Inherit. Metab. Dis. 37 (6), 979–990. 10.1007/s10545-014-9715-6 24810369 PMC4206772

[B22] Hendriksz C. J. Parini R. AlSayed M. D. Raiman J. Giugliani R. Solano Villarreal M. L. (2016). Long-term endurance and safety of elosulfase alfa enzyme replacement therapy in patients with Morquio A syndrome. Mol. Genet. Metab. 119 (1-2), 131–143. 10.1016/j.ymgme.2016.05.018 27380995

[B23] Huang Y. Chen P. Peng B. Liao R. Huang H. Huang M. (2023). The top 100 most cited articles on triple-negative breast cancer: a bibliometric analysis. Clin. Exp. Med. 23 (2), 175–201. 10.1007/s10238-022-00800-9 35416524

[B24] Hunter C. (1917). A rare disease in two brothers. Proc. R. Soc. Med. 10, 104–116. 10.1177/003591571701001833 19979883 PMC2018097

[B25] Karsan R. B. Powell A. G. Nanjaiah P. Mehta D. Valtzoglou V. (2019). The top 100 manuscripts in emergency cardiac surgery. Potential role in cardiothoracic training. A bibliometric analysis. Ann. Med. Surg. (Lond) 43, 5–12. 10.1016/j.amsu.2019.05.002 31193454 PMC6531840

[B26] Karslı B. Tekin S. B. (2021). The top 100 most-cited articles on ankle arthroscopy: bibliometric analysis. J. Foot Ankle Surg. 60 (3), 477–481. 10.1053/j.jfas.2020.08.028 33518508

[B27] Kendall R. L. Holian A. (2021). The role of lysosomal ion channels in lysosome dysfunction. Inhal. Toxicol. 33 (2), 41–54. 10.1080/08958378.2021.1876188 33627009

[B28] Khan S. A. Peracha H. Ballhausen D. Wiesbauer A. Rohrbach M. Gautschi M. (2017). Epidemiology of mucopolysaccharidoses. Mol. Genet. Metab. 121 (3), 227–240. 10.1016/j.ymgme.2017.05.016 28595941 PMC5653283

[B29] Kobayashi H. (2019). Recent trends in mucopolysaccharidosis research. J. Hum. Genet. 64 (2), 127–137. 10.1038/s10038-018-0534-8 30451936

[B30] Kreutzer J. S. Agyemang A. A. Weedon D. Zasler N. Oliver M. Sorensen A. A. (2017). The top 100 cited neurorehabilitation papers. NeuroRehabilitation 40 (2), 163–174. 10.3233/NRE-161415 28222551

[B31] Liu P. C. Lu Y. Lin H. H. Yao Y. C. Wang S. T. Chang M. C. (2022). Classification and citation analysis of the 100 top-cited articles on adult spinal deformity since 2011: a bibliometric analysis. J. Chin. Med. Assoc. 85 (3), 401–408. 10.1097/jcma.0000000000000642 34698695 PMC12755497

[B32] Liu R. Peng B. Yuan J. Hu J. Yang J. Shan N. (2024). Research on stem cell therapy for spinal cord injury: a bibliometric and visual analysis from 2018-2023. Front. Genet. 15, 1327216. 10.3389/fgene.2024.1327216 38380424 PMC10877028

[B33] Mainwaring A. Bullock N. Ellul T. Hughes O. Featherstone J. (2020). The top 100 most cited manuscripts in bladder cancer: a bibliometric analysis (review article). Int. J. Surg. 75, 130–138. 10.1016/j.ijsu.2020.01.128 31991242

[B34] McGill J. J. Inwood A. C. Coman D. J. Lipke M. L. de Lore D. Swiedler S. J. (2010). Enzyme replacement therapy for mucopolysaccharidosis VI from 8 weeks of age--a sibling control study. Clin. Genet. 77 (5), 492–498. 10.1111/j.1399-0004.2009.01324.x 19968667

[B35] Muenzer J. (2014). Early initiation of enzyme replacement therapy for the mucopolysaccharidoses. Mol. Genet. Metab. 111 (2), 63–72. 10.1016/j.ymgme.2013.11.015 24388732

[B36] Muenzer J. Beck M. Eng C. M. Giugliani R. Harmatz P. Martin R. (2011a). Long-term, open-labeled extension study of idursulfase in the treatment of Hunter syndrome. Genet. Med. 13 (2), 95–101. 10.1097/GIM.0b013e3181fea459 21150784

[B37] Muenzer J. Beck M. Giugliani R. Suzuki Y. Tylki-Szymanska A. Valayannopoulos V. (2011b). Idursulfase treatment of Hunter syndrome in children younger than 6 years: results from the Hunter Outcome Survey. Genet. Med. 13 (2), 102–109. 10.1097/GIM.0b013e318206786f 21233716

[B38] Muenzer J. Gucsavas-Calikoglu M. McCandless S. E. Schuetz T. J. Kimura A. (2007). A phase I/II clinical trial of enzyme replacement therapy in mucopolysaccharidosis II (Hunter syndrome). Mol. Genet. Metab. 90 (3), 329–337. 10.1016/j.ymgme.2006.09.001 17185020

[B39] Muenzer J. Wraith J. E. Beck M. Giugliani R. Harmatz P. Eng C. M. (2006). A phase II/III clinical study of enzyme replacement therapy with idursulfase in mucopolysaccharidosis II (Hunter syndrome). Genet. Med. 8 (8), 465–473. 10.1097/01.gim.0000232477.37660.fb 16912578

[B40] Muenzer J. Wraith J. E. Clarke L. A. International Consensus Panel on Management and Treatment of Mucopolysaccharidosis I (2009). Mucopolysaccharidosis I: management and treatment guidelines. Pediatrics 123 (1), 19–29. 10.1542/peds.2008-0416 19117856

[B41] Nagpal R. Goyal R. B. Priyadarshini K. Kashyap S. Sharma M. Sinha R. (2022). Mucopolysaccharidosis: a broad review. Indian J. Ophthalmol. 70 (7), 2249–2261. 10.4103/ijo.IJO_425_22 35791104 PMC9426054

[B42] Okuyama T. Eto Y. Sakai N. Nakamura K. Yamamoto T. Yamaoka M. (2021). A phase 2/3 trial of pabinafusp alfa, IDS fused with anti-human transferrin receptor antibody, targeting neurodegeneration in MPS-II. Mol. Ther. 29 (2), 671–679. 10.1016/j.ymthe.2020.09.039 33038326 PMC7854283

[B43] Parenti G. Medina D. L. Ballabio A. (2021). The rapidly evolving view of lysosomal storage diseases. EMBO Mol. Med. 13 (2), e12836. 10.15252/emmm.202012836 33459519 PMC7863408

[B44] Pinto R. Caseiro C. Lemos M. Lopes L. Fontes A. Ribeiro H. (2004). Prevalence of lysosomal storage diseases in Portugal. Eur. J. Hum. Genet. 12 (2), 87–92. 10.1038/sj.ejhg.5201044 14685153

[B45] Platt F. M. (2018). Emptying the stores: lysosomal diseases and therapeutic strategies. Nat. Rev. Drug Discov. 17 (2), 133–150. 10.1038/nrd.2017.214 29147032

[B46] Polgreen L. E. Kunin-Batson A. Rudser K. Vehe R. K. Utz J. J. Whitley C. B. (2017). Pilot study of the safety and effect of adalimumab on pain, physical function, and musculoskeletal disease in mucopolysaccharidosis types I and II. Mol. Genet. Metab. Rep. 10, 75–80. 10.1016/j.ymgmr.2017.01.002 28119823 PMC5238608

[B47] Puckett Y. Mallorga-Hernández A. Montaño A. M. (2021). Epidemiology of mucopolysaccharidoses (MPS) in United States: challenges and opportunities. Orphanet J. Rare Dis. 16 (1), 241. 10.1186/s13023-021-01880-8 34051828 PMC8164808

[B48] Regenxbio (2023). Additional positive interim data from phase I/II/III CAMPSIITE™ trial of REGENXBIO's RGX-121 for the treatment of MPS II (hunter syndrome) presented at 19th annual WORLDSymposiumTM. Avaliable at: https://regenxbio.gcs-web.com/news-releases/news-release-details/additional-positive-interim-data-phase-iiiiii-campsiitetm-trial .

[B49] Regenxbio (2024). REGENXBIO announces pivotal trial of RGX-121 for the treatment of MPS II achieves primary endpoint. Avaliable at: https://www.prnewswire.com/news-releases/regenxbio-announces-pivotal-trial-of-rgx-121-for-the-treatment-of-mps-ii-achieves-primary-endpoint-302056283.html .

[B50] Rintz E. Podlacha M. Cyske Z. Pierzynowska K. Węgrzyn G. Gaffke L. (2023). Activities of (Poly)phenolic antioxidants and other natural autophagy modulators in the treatment of sanfilippo disease: remarkable efficacy of resveratrol in cellular and animal models. Neurotherapeutics 20 (1), 254–271. 10.1007/s13311-022-01323-7 36344724 PMC10119361

[B51] Rodgers N. J. Kaizer A. M. Miller W. P. Rudser K. D. Orchard P. J. Braunlin E. A. (2017). Mortality after hematopoietic stem cell transplantation for severe mucopolysaccharidosis type I: the 30-year University of Minnesota experience. J. Inherit. Metab. Dis. 40 (2), 271–280. 10.1007/s10545-016-0006-2 28054207

[B52] Scott H. S. Bunge S. Gal A. Clarke L. A. Morris C. P. Hopwood J. J. (1995). Molecular genetics of mucopolysaccharidosis type I: diagnostic, clinical, and biological implications. Hum. Mutat. 6 (4), 288–302. 10.1002/humu.1380060403 8680403

[B53] Shapiro E. G. Eisengart J. B. (2021). The natural history of neurocognition in MPS disorders: a review. Mol. Genet. Metab. 133 (1), 8–34. 10.1016/j.ymgme.2021.03.002 33741271

[B54] Snyder E. Y. Taylor R. M. Wolfe J. H. (1995). Neural progenitor cell engraftment corrects lysosomal storage throughout the MPS VII mouse brain. Nature 374 (6520), 367–370. 10.1038/374367a0 7885477

[B55] Sugimoto C. R. Robinson-Garcia N. Murray D. S. Yegros-Yegros A. Costas R. Larivière V. (2017). Scientists have most impact when they're free to move. Nature 550 (7674), 29–31. 10.1038/550029a 28980663

[B56] Tardieu M. Zérah M. Husson B. de Bournonville S. Deiva K. Adamsbaum C. (2014). Intracerebral administration of adeno-associated viral vector serotype rh.10 carrying human SGSH and SUMF1 cDNAs in children with mucopolysaccharidosis type IIIA disease: results of a phase I/II trial. Hum. Gene Ther. 25 (6), 506–516. 10.1089/hum.2013.238 24524415

[B57] Taylor M. Khan S. Stapleton M. Wang J. Chen J. Wynn R. (2019). Hematopoietic stem cell transplantation for mucopolysaccharidoses: past, present, and future. Biol. Blood Marrow Transpl. 25 (7), e226–e246. 10.1016/j.bbmt.2019.02.012 PMC6615945 30772512

[B58] Tillo M. Lamanna W. C. Dwyer C. A. Sandoval D. R. Pessentheiner A. R. Al-Azzam N. (2022). Impaired mitophagy in Sanfilippo a mice causes hypertriglyceridemia and brown adipose tissue activation. J. Biol. Chem. 298 (8), 102159. 10.1016/j.jbc.2022.102159 35750212 PMC9364035

[B59] Wang W. Wang H. Yao T. Li Y. Yi L. Gao Y. (2023). The top 100 most cited articles on COVID-19 vaccine: a bibliometric analysis. Clin. Exp. Med. 23 (6), 2287–2299. 10.1007/s10238-023-01046-9 36939968 PMC10026222

[B60] Wood S. R. Bigger B. W. (2022). Delivering gene therapy for mucopolysaccharide diseases. Front. Mol. Biosci. 9, 965089. 10.3389/fmolb.2022.965089 36172050 PMC9511407

[B61] Wraith J. E. Scarpa M. Beck M. Bodamer O. A. De Meirleir L. Guffon N. (2008). Mucopolysaccharidosis type II (Hunter syndrome): a clinical review and recommendations for treatment in the era of enzyme replacement therapy. Eur. J. Pediatr. 167 (3), 267–277. 10.1007/s00431-007-0635-4 18038146 PMC2234442

[B62] Xu J. Núñez G. (2023). The NLRP3 inflammasome: activation and regulation. Trends Biochem. Sci. 48 (4), 331–344. 10.1016/j.tibs.2022.10.002 36336552 PMC10023278

[B63] Zhang Y. Hu M. Wang J. Wang P. Shi P. Zhao W. (2022). A bibliometric analysis of personal protective equipment and COVID-19 researches. Front. Public Health 10, 855633. 10.3389/fpubh.2022.855633 35570977 PMC9099374

[B64] Zhang Y. Rong L. Wang Z. Zhao H. (2023). The top 100 most cited articles in helical tomotherapy: a scoping review. Front. Oncol. 13, 1274290. 10.3389/fonc.2023.1274290 37916164 PMC10616822

[B65] Zhou J. J. Koltz M. T. Agarwal N. Tempel Z. J. Kanter A. S. Okonkwo D. O. (2017). 100 most influential publications in scoliosis surgery. Spine (Phila Pa 1976) 42 (5), 336–344. 10.1097/BRS.0000000000001860 28245207

[B66] Zhou Y. Liu M. Huang X. Liu Z. Sun Y. Wang M. (2023). Emerging trends and thematic evolution of immunotherapy for glioma based on the top 100 cited articles. Front. Oncol. 13, 1307924. 10.3389/fonc.2023.1307924 38293697 PMC10825959

[B67] Zhu H. Zhang Z. (2021). Emerging trends and research foci in cataract genes: a bibliometric and visualized study. Front. Genet. 12, 610728. 10.3389/fgene.2021.610728 34434212 PMC8381374

[B68] Zhu Y. Zhang C. Wang J. Xie Y. Wang L. Xu F. (2021). The top 100 highly cited articles on anterior cruciate ligament from 2000 to 2019: a bibliometric and visualized analysis. Orthop. Traumatol. Surg. Res. 107 (8), 102988. 10.1016/j.otsr.2021.102988 34146752

